# A Self-Adhesive Elastomeric Wound Scaffold for Sensitive Adhesion to Tissue

**DOI:** 10.3390/polym11060942

**Published:** 2019-05-31

**Authors:** Silviya Boyadzhieva, Katharina Sorg, Martin Danner, Sarah C. L. Fischer, René Hensel, Bernhard Schick, Gentiana Wenzel, Eduard Arzt, Klaus Kruttwig

**Affiliations:** 1 INM-Leibniz Institute for New Materials, Campus D2.2, D-66123 Saarbrücken, Germany; Silviya.Boyadzhieva@leibniz-inm.de (S.B.); martin.danner93@googlemail.com (M.D.); sarahclfischer@gmail.com (S.C.L.F.); Rene.Hensel@leibniz-inm.de (R.H.); Eduard.Arzt@leibniz-inm.de (E.A.); 2 Department of Materials Science and Engineering, Saarland University, Campus D2.2, D-66123 Saarbrücken, Germany; 3 Department of Otorhinolaryngology, Saarland University Medical Center, D-66421 Homburg, Germany; Katharina.Sorg@uks.eu (K.S.); Bernhard.Schick@uks.eu (B.S.); Gentiana.Wenzel@uks.eu (G.W.)

**Keywords:** PDMS, soft skin adhesive, scaffold material, protein coating, PSA, tympanic membrane, scaffold, cells, wound dressing, self-adhesive

## Abstract

Pressure sensitive adhesives based on silicone materials are used particularly for skin adhesion, e.g., the fixation of electrocardiogram (ECG) electrodes or wound dressings. However, adhesion to sensitive tissue structures is not sufficiently addressed due to the risk of damage or rupture. We propose an approach in which a poly-(dimethylsiloxane) (PDMS)-based soft skin adhesive (SSA) acts as cellular scaffold for wound healing. Due to the intrinsically low surface free energy of silicone elastomers, functionalization strategies are needed to promote the attachment and spreading of eukaryotic cells. In the present work, the effect of physical adsorption of three different proteins on the adhesive properties of the soft skin adhesive was investigated. Fibronectin adsorption slightly affects adhesion but significantly improves the cellular interaction of L929 murine fibroblasts with the polymeric surface. Composite films were successfully attached to explanted tympanic membranes. This demonstrates the potential of protein functionalized SSA to act as an adhesive scaffold in delicate biomedical applications.

## 1. Introduction

Silicone-based pressure sensitive adhesives (PSA) are widely used as adhesives to wounded skin as they adhere with small applied pressure and after short contact time. Classical application fields include the fixation of electrocardiogram (ECG) patches or of wearable electronic devices [1,2,3]. Silicones are a versatile class of polymeric materials exhibiting a low surface free energy and high flexibility [4]. One of the most extensively used silicone elastomers is poly-(dimethylsiloxane) (PDMS) with broad application as a skin adhesive [1,5,6,7,8]. Recently, a subclass of PDMS, so called soft skin adhesives (SSAs), has been introduced, in particular for gentle skin applications. SSAs have the ability to adhere to rough surfaces including wet skin, exhibit a high water vapor permeability, and good compatibility with pharmaceutical compounds [7,8,9,10]. The gentle attachment and detachment mechanisms enable a reasonable balance between secure adhesion and atraumatic removal. However, due to the hydrophobic nature of PDMS, cellular adhesion and spreading are markedly impaired, making appropriate surface modifications necessary. Cell-material interactions can be improved, e.g., by physical adsorption of proteins or oxygen plasma treatment [11,12,13,14]. Physical protein adsorption is primarily based on molecular interactions such as electrostatic, hydrophobic, van der Waals, or hydrogen bonding [15]. These non-covalent bonds are often weak, compared to covalent bonding, especially in an aqueous environment [16,17].

Due to the virtual absence of functional groups for covalent immobilization in PDMS, surface treatments are commonly applied to introduce silanol groups [18,19]. This includes ultraviolet light (UV), corona, or plasma application. These treatments are frequently performed as an initial process for more complex, durable surface modifications [18,20,21]. However, the adhesive performance of the soft skin adhesive can be significantly diminished after treatment with oxygen- or air plasma, highlighting the need for alternative modification methods [22]. To promote cellular attachment and spreading, fibronectin and fibrinogen have been applied on different substrates, including PDMS [23,24,25]. In contrast, a heparin or albumin coating of biomaterials prevents platelet activation and can inhibit bacterial adhesion [26,27]. So far, it remains an open question to what extent different protein coatings on polymeric surfaces influence their adhesive properties.

In the present paper, we analyze the adhesive properties of single layers and composite films of PDMS, Sylgard 184 and SSA MG 7-9800, by normal adhesion (tack) and peel test, as a function of protein surface functionalization. Additionally, polymeric films were attached to explanted murine tympanic membranes to demonstrate their potential to act as a novel wound dressing. The tympanic membrane, a thin trilamellar structure with an essential function in acoustic wave transmission, can be affected pathophysiologically by perforations [28]. For therapeutic purposes, the application of a synthetic material combining scaffold abilities with adhesive function would be beneficial [29,30].

## 2. Materials and Methods

### 2.1. Preparation of Thin Elastomeric Films

**Figure 1:**
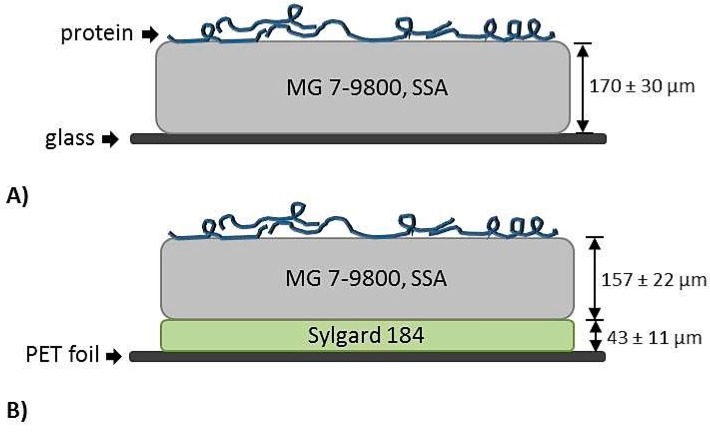
Schematic representation of the polymeric samples: soft skin adhesive (SSA) MG 7-9800 was applied on a glass surface (**A**) or manufactured as a composite film on a PET foil (**B**).

Soft Skin Adhesive silicone elastomer (MG 7-9800, SSA) was obtained from Dow Corning (Auburn, MI, USA). Sylgard 184 (Dow Corning, Auburn, MI, USA) was used as a control polymer for the investigation of the protein adsorption in vitro as well as for manufacturing of the composite films. The two components of each PDMS were mixed in a ratio of 1:1 (for SSA) and 10:1 (for Sylgard 184) weight parts and homogenized for 3 min under vacuum at 2350 rpm in a SpeedMixer (DAC600.2 VAC-P, Hauschild Engineering, Hamm, Germany). An automatic doctor blade application machine (AFA-IV, MTI Corporation, Richmond, CA, USA) was used to manufacture single SSA or Sylgard 184 layers (on glass plates) and composite films (on PET foil) consisting of a Sylgard 184 backing layer and a SSA top layer, as shown in Figure 1 [31]. To produce the composite films, a Sylgard 184 layer was applied on an isopropanol cleaned PET foil and cured in an oven (Heraeus Vacutherm, Thermo Fisher Scientific, Waltham, MA, USA) at 95 °C for one hour. After curing, the side edges of the Sylgard 184 film were removed, the top layer of SSA was applied on it and subsequently cured. The wet thickness of the single SSA or Sylgard 184 layers was chosen to be 300 µm and for the composite films: 100 µm for Sylgard 184 and 320 µm for SSA. The thickness of the cured polymeric layers was determined with an optical microscope (VHX-2000, Keyence, Osaka, Japan). Measured values are indicated in Figure 1. For the MG 7-9800 film, a thickness of 170 ± 30 µm was determined, as shown in Figure 1A. The thickness of the composite film was 43 ± 11 µm for Sylgard 184 and 157 ± 22 µm for SSA, as shown in Figure 1B.

### 2.2. Protein Adsorption

Three different proteins were used for surface treatment: bovine serum albumin (BSA) (Carl ROTH, Karlsruhe, Germany), fibronectin from bovine plasma (FN) (Sigma Aldrich, Taufkirchen, Germany) and fibrinogen from bovine plasma (FG) (Sigma Aldrich, Taufkirchen, Germany). For cell culture experiments and tack tests, SSA films were incubated with 10 µg/mL of the proteins dissolved in ddH_2_O for one hour at 37 °C and 5% CO_2_. SSA films were incubated in ddH_2_O as a control. After the incubation time, samples were washed and subsequently dried for at least 30 min.

The amount of physically adsorbed protein was determined with a microBCA protein assay kit (Thermo Fisher Scientific, Waltham, MA, USA). Circular areas fitting into a single well (approximately 9.4 cm^2^) of a 6 well plate (Greiner Bio-One, CELLSTAR, Frickenhausen, Germany) were excised from the films (including the PET foil) and placed into the wells. The protein solutions were prepared in ddH_2_O and the exact protein concentrations were determined with the microBCA test (three independent prepared solutions): 11.9 ± 3.7 µg/mL for albumin; 10.3 ± 4.8 µg/mL for fibronectin, and 8.3 ± 3.6 µg/mL for fibrinogen. To a single well containing a polymeric sample, 3 mL of the individual protein solution was added and incubated for one hour at 37 °C and 5% CO_2_. After removing the protein solutions, the samples were washed three times with deionized water to remove excess proteins. Next, 1 mL of 1% sodium dodecyl sulfate (SDS) prepared in ddH_2_O was added to each well for 20 min at 37 °C and 5% CO_2_. Then, 0.5 mL of each SDS solution was transferred to a reaction tube (Eppendorf, Hamburg, Germany) containing 0.5 mL microBCA working reagent. The tubes were placed in a thermomixer (Eppendorf, Hamburg, Germany) for 1 h at 60 °C and 300 rpm. Subsequently, the measurements were performed with a microplate reader (absorbance at 562 nm, SpectraMax 190, Molecular Devices, San Jose, CA, USA). In some experiments, the intensity of the control samples was higher than the staining of the protein treated films and was considered as artifacts. For this reason, these experiments were excluded from the final analysis. The staining of the control samples could be significantly reduced by washing the polymeric surface with water prior to protein deposition, indicating the importance of a pre-equilibration phase of the samples before protein treatment. Values are reported in ng/cm^2^ in the text and corresponding figure.

### 2.3. Cell Culture Experiments and Staining

The biological examination of the SSA films (protein treated, immersed in water, pristine) was performed using the murine fibroblast cell line L929. Cells were cultured in Roswell Park Memorial Institute (RPMI) basal medium, supplemented with 10% fetal bovine serum and penicillin/streptomycin at 37 °C and 5% CO_2_. The cultured cells were passaged according to standard procedures with Accutase (Capricorn Scientific, Ebsdorfergrund, Germany) and cell number was determined using a Neubauer chamber. Next, 6 × 10^4^ cells were seeded directly on the polymers coated with fibronectin, fibrinogen, albumin and pristine polymers in 24 well plates. After a culture period of 48 h, phase contrast images were acquired before fixation with 4% paraformaldehyde (PFA, Electron Microscopy Sciences, Hatfield, PA, USA) for 25 min at room temperature. For the staining experiments, unspecific binding sites were blocked by incubating each well with 5% bovine serum albumin (BSA), 0.2% Triton X-100, PBS for 60 min at room temperature (RT). For visualization of the actin cytoskeleton, phalloidin conjugated Alexa-488 solution (1:160, Thermo Fisher Scientific, Waltham, MA, USA) was incubated for 3 h at room temperature or overnight at 4 °C. Hoechst Dye 33342 (1:1000, Sigma Aldrich, Taufkirchen, Germany) was used as a nuclear dye. For detection of focal adhesion sites, anti-phospho-FAK^Tyr397^ monoclonal antibody (1:2000, Abcam, Cambridge, UK), followed by incubation with a secondary antibody (1:1000, Alexa Fluor 594 goat anti-rabbit, Thermo Fisher Scientific, Waltham, MA, USA) was used. The samples were embedded with mounting medium (Aqua-Poly/Mount, Polysciences Europe GmbH, Hirschberg, Germany) and images were acquired with a fluorescence microscope (Leica, Wetzlar, Germany), using the software Leica Application Suite X and post-processed with Image J. Exposure time and further settings (intensity, gain) were kept constant during acquisition. The post-processing was limited to the combination of the different fluorescence channels and adjustment of brightness values and contrast. The cellular area was calculated on phase contrast images using ImageJ.

### 2.4. Analysis of Adhesion Properties: Peel and Tack Test

Protein treated elastomeric films (manufactured as described in Section 2.2) and two control conditions (incubated in deionized water and pristine) were investigated. Samples were treated with protein or immersed in ddH_2_O for one hour, washed with deionized water, and stored for at least 24 h at room temperature allowing the surface to dry. The determination of tack and peel properties of the adhesive films was performed with a custom-built setup (macroscopic adhesion measurement device MAD, as shown in Figure S1a, see Supplementary Materials) [22,32]. Single layers of SSA on glass substrate or composite films on PET foil with an area of approximately 5 cm^2^ were affixed to glass slides with UV-glue (Bohle, Haan, Germany). The tack tests were performed with two flat, rigid glass substrates exhibiting different surface roughness. The “smooth” glass substrate exhibited an area of 3.2 mm^2^ and a mean peak-to-valley roughness of R_z_ = 0.12 ± 0.004 µm. For the manufacturing of the “rough” substrate, a small slice of frosted glass (Carl Roth, Karlsruhe, Germany) was mechanically cut and ground to attain a circular shape (area 6.07 mm^2^, R_z_ = 2.055 ± 0.017 µm). The machine compliance (*C* = 0.12 µm/mN) was determined and taken into account to correct the displacement during the adhesion measurements.

The samples were affixed to a holder and mounted on a tilting table allowing precise adjustment of the two surfaces. Attachment and detachment processes were observed with two cameras. The film was brought into contact with the substrate surface until a compressive preload stress $\sigma_{0}$ of 13 ± 5 kPa was achieved. After a hold time of 1 s, the sample was retracted. The approach and detachment velocity were set to 30 and 10 µm/s, respectively. During the entire process of bond formation and retraction, the sample position s and the normal force F were recorded. Two parameters were chosen to describe the adhesive properties of the films—the maximum detachment force per contact area *A* or pull of stress, $\sigma_{max}=\max\left(\frac{F}{A}\right)$, and the work of separation $W_{sep}=\int_{s_{0}}^{s_{end}}\sigma ds$ ($s_{0}$: displacement at the start of detachment, $s_{end}$: displacement at completed detachment).

The set up was further modified to allow an investigation of the peel behavior of the polymeric films, as shown in Figure S1b. The measurements were limited to a flat square glass substrate with an area of 6 cm^2^ exhibiting similar roughness values to the rough glass used for the tack analysis. Only films treated with fibronectin and control conditions were selected for these experiments. Composite films (thickness: 43 ± 11 µm for Sylgard 184 and 157 ± 22 µm for SSA) were manufactured on PET foil. Strips with dimensions of about 2 cm × 0.5 cm were cut out and removed from the PET foil. The shorter side of the strip was affixed to an aluminum holder located on the load cell. A part of the film (area of approximately 1 cm × 0.5 cm) was brought into contact with the substrate. The strips were peeled off under an initial angle *Ѳ* ≈ 90° with a constant velocity of 50 µm/s. A time–force curve was recorded and the maximum peel force was used for further analysis.

### 2.5. Peel Tests on Explanted Mouse Tympanic Membrane

Peel tests on explanted mouse tympanic membranes were performed with a custom made peel tester to measure the adhesive strength of composite films as principally shown by Bundy et al. [33]. The different steps of attachment, adhesion, and peeling of the SSA-based patches are shown in Figure S2. The petrosal bone, including the tympanic cavity of mice, was prepared from 6 to 8 week old CBA/J mice. Sacrificing and preparation of mice for scientific purposes was in full accordance with the German Animal Welfare Law. The Animal Welfare Officer of the Saarland University, Germany had been informed in advance and the euthanasia methods were fully appropriate. Proper procedures were in place for minimizing discomfort, distress, and pain of experimental animals (mice). The tympanic sulcus was affixed to a glass substrate using a two-component methyl methacrylate (Technovit 4004, Kulzer Technik, Germany) while ensuring free oscillation of the eardrum. After curing, the glass substrate was mounted to a load cell (ME-Meßsysteme GmbH, Hennigsdorf, Germany, #KD34s 0.25N). Under visual control, adhesive films were cut in circular pieces with a diameter of 1.2 mm and applied to the tympanic membrane. They exhibited a thickness of 43 ± 11 µm for the Sylgard 184 backing layer and 157 ± 22 µm for the SSA top layer. Afterwards, the films were manually peeled off using tweezers, as shown in Figure 7A and Figure S2. As a reference material, a Sylgard 184 film with a thickness of approximately 40 µm was used. For comparison, commercially available silicone strips for clinical applications (bess pro, BM201001, Berlin, Germany) were tested in wet and dry conditions.

### 2.6. Statistical Analysis

For statistical analysis, SPSS (IBM SPSS Statistics 19) was used. For normal distributed data, a one-way analysis of variance (one-way ANOVA) was performed, followed by a Levene’s test to estimate homogeneity of variance. The Bonferroni and Dunnett test was used in case of variance equality and Games–Howell in case of variance inequality. Whenever the data was not normal distributed, a Kruskal–Wallis H test was used as a non-parametric method. A significance level of *p* = 0.05 was chosen for all tests. In all figures the error bars represent the standard deviation.

## 3. Results and Discussion

### 3.1. Physical Adsorption of Proteins on PDMS Surfaces

**Figure 2:**
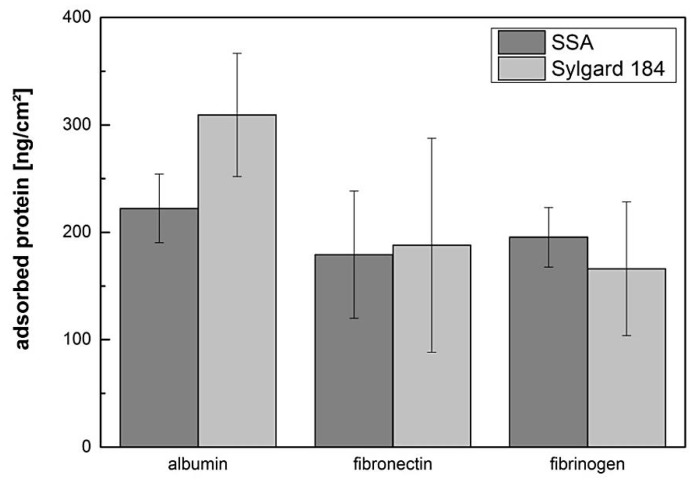
Protein adsorption on polymeric surfaces: Sylgard 184 and SSA single layers manufactured on PET foil were incubated in solutions of albumin (11.9 ± 3.7 µg/mL), fibronectin (10.3 ± 4.8 µg/mL), and fibrinogen (8.3 ± 3.6 µg/mL) for one hour at 37 °C. The amount of physically adsorbed protein was determined with microBCA spectrophotometric analysis and normalized to the surface area. Number of independently performed experiments n = 3. Statistical analysis revealed no difference while comparing SSA to Sylgard 184.

The adsorption ability of proteins on PDMS surfaces depends on various environmental factors, including incubation time, ionic strength, protein concentration, and surface free energy [16,34]. To determine the amount of bound protein, the physical adsorption of albumin, fibrinogen, and fibronectin onto the surface of SSA and Sylgard 184 was investigated, as shown in Figure 2.

After the incubation period, the highest protein amount of 309 ± 57 ng/cm^2^ was detected on the Sylgard 184 films coated with albumin. On the SSA films, an amount of 222 ± 32 ng/cm^2^ was observed, as shown in Figure 2. On films incubated with fibrinogen and fibronectin, comparable protein concentrations were measured (fibronectin: 179 ± 59 ng/cm^2^ on SSA versus 188 ± 100 ng/cm^2^ on Sylgard 184; fibrinogen: 195 ± 28 ng/cm^2^ on SSA versus 166 ± 62 ng/cm^2^ on Sylgard 184). The measured protein amount was in accordance to values reported in literature [17,23,35]. For example, Toworfe et al. reported an amount of 122 ng/cm^2^ after incubating for 1 h with 2.5 µg/mL fibronectin and 480 ng/cm^2^ after incubating with 10 µg/mL [23]. Statistical analysis for all three proteins revealed no significant difference while comparing Sylgard 184 to SSA, as shown in Figure 2. Using fluorescence conjugated albumin, we found previously that the protein adsorption capacity is comparable for Sylgard 184 and SSA MG 7-9800, which could be verified in the current study [22].

### 3.2. Cellular Adhesion and Spreading of Fibroblasts on Protein Functionalized PDMS Surfaces

**Figure 3:**
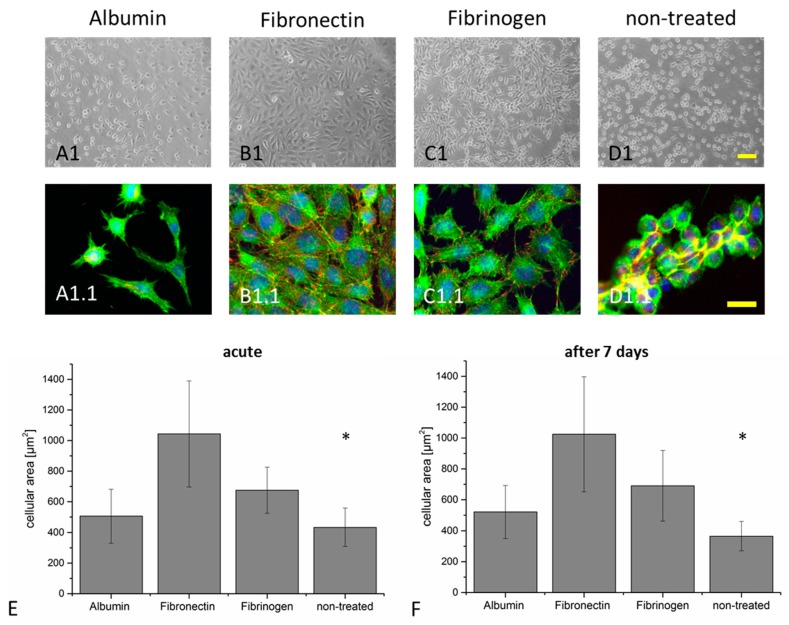
Cellular response to the protein coating on the elastomer films. L929 murine fibroblasts were cultured on SSA films coated with albumin (**A1**, **A1.1**), fibronectin (**B1**, **B1.1**), fibrinogen (**C1**, **C1.1**), and non-treated (pristine) polymeric surface (**D1**, **D1.1**). Phase contrast pictures were acquired after a culture period of 48 h (**A1**, **B1**, **C1**, **D1**). Additionally, the cells were stained with an anti phospho-FAKTyr^397^ antibody to visualize focal adhesion contacts (red). The actin cytoskeleton (green) and cellular nuclei (blue) were stained (**A1.1**, **B1.1**, **C1.1**, and **D1.1**). Cellular spreading was determined on films that have been directly used for cell culture (**E**) and on films stored for seven days in ddH_2_O at 37 °C before seeding the cells (**F**) (see also Figure S3). Number of independent experiments: n = 3 for the fluorescence analysis; n = 4 for the determination of the cellular area. Scale bar in A1, B1, C1, D1 = 100 µm; scale bar in A1.1, B1.1, C1.1, D1.1 = 25 µm. At a specific time point, statistically significant differences were detected between all conditions. Furthermore, no significant difference could be detected while comparing both time points (acute versus seven days, indicated by an asterisk, *p* ≤ 0.05).

To investigate if the protein amounts deposited on the elastomeric surface are sufficient to promote cellular adhesion and spreading, cell biological experiments with L929 fibroblasts were performed. Without any protein coverage, the cells were only weakly attached to the surface after 48 h, as shown in Figure 3D1. They presented minor extensions of lamellipodia and the cellular morphology was dominated by a compact, round appearance, as shown in Figure 3D1.

In order to visualize cell–substrate interactions, the presence of focal adhesion contacts to the substrate was verified with an antibody directed against focal adhesion kinase (FAK) phosphorylated at amino acid Tyr^397^, as shown in Figure 3D1.1. As expected from the phase contrast images, very few FAK^Tyr397^ positive focal adhesive contacts were present in the pristine conditions, as shown in Figure 3D1.1. The morphology of cells cultured on albumin treated surfaces was similar to the pristine conditions, mostly dominated by cells weakly adhered to the surface, with few FAK^Tyr397^ positive focal adhesion contacts, as shown in Figure 3A1,A1.1. Coating with fibrinogen and fibronectin promoted adhesion and spreading of the L929 cells on the SSA surface with formation of distinct FAK^Tyr397^ positive focal adhesion contacts as well, as shown in Figure 3B1,C1,B1.1,C1.1.

Surface functionalization with albumin represents a medically relevant treatment to prevent especially bacterial adherence and to decrease in vitro platelet adhesion on polymeric materials and aortic prostheses [27,36]. Albumin coating of different materials has also been used to promote the adherence of several different eukaryotic cell types, including bone-marrow-derived mesenchymal stem cells and MC3T3-E1 osteoblast-like cells [37,38,39]. Importantly, cellular adhesion properties are strongly dependent on the albumin surface concentration and can also be influenced by UV treatment [37,39,40]. Additionally, to improve cellular adhesion, biomaterial surfaces can be coated with fibronectin or fibrinogen [13,41,42]. Experimentally, it was demonstrated that stent coating with these two proteins promote re-endothelialization after percutaneous coronary intervention [25].

Quantification of cellular spreading and adhesion is a sensitive and frequently applied technique to assess surface properties [23,43,44]. Here, the cellular area was determined to specifically investigate the effect of the different proteins on the cellular adhesion and spreading performance. We included two different conditions into this study—polymers were functionalized and used directly for cellular investigation (condition: acute), as shown in Figure 3A–E.

Secondly, the cells were seeded on polymers that had been immersed in water for seven days (condition: seven days), as shown in Figure 3F and Figure S3. With a mean cellular area of 1044 ± 347 µm^2^ (acute) and 1025 ± 373 µm^2^ (after seven days), the spreading of the cells was significantly higher for cells cultured on fibronectin compared to all other tested conditions (fibrinogen: 676 ± 151 µm^2^ (acute) and 691 ± 229 µm^2^ (after seven days); albumin: 506 ± 176 µm^2^ (acute) and 521 ± 171 µm^2^ (after seven days); pristine: 434 ± 125 µm^2^ (acute) and 365 ± 95 µm^2^ (after seven days), as shown in Figure 3E,F. The cellular contact to the fibronectin treated surface was strong without the occurrence of floating cells, even after brief mechanical stimulation (data not shown). Coating with fibronectin resulted in homogeneous cellular coverage of the entire polymer surface in contrast to the pristine condition, where only partial surface coverage was achieved, as shown in Figure 3E. Interestingly, the cellular morphology, regarding spreading and surface coverage, demonstrated no significant difference between acute and seven days of storage except for the non-treated condition. This indicates the presence of a sufficient protein amount to support cellular function even after seven days of storage, as shown in Figure 3E,F. It was already shown in the literature, that a wide range of protein concentrations can be used for coating different substrates [17,23,41,45] Toworfe et al. investigated the effect of physical adsorption of two different protein concentrations onto a PDMS surface in detail [23]. After incubating PDMS with a solution of 10 or 2.5 µg/mL led to a protein surface density of 480 and 122 ng/mL, respectively. Therein, no remarkable difference in the cellular area of MC3T3-E1 osteoblast cells, cultured on pristine PDMS that have been coated with either 10 or 2.5 µg/mL could be observed.

### 3.3. Tack Analysis of Adhesion Performance on Protein Functionalized SSA Films

**Figure 4:**
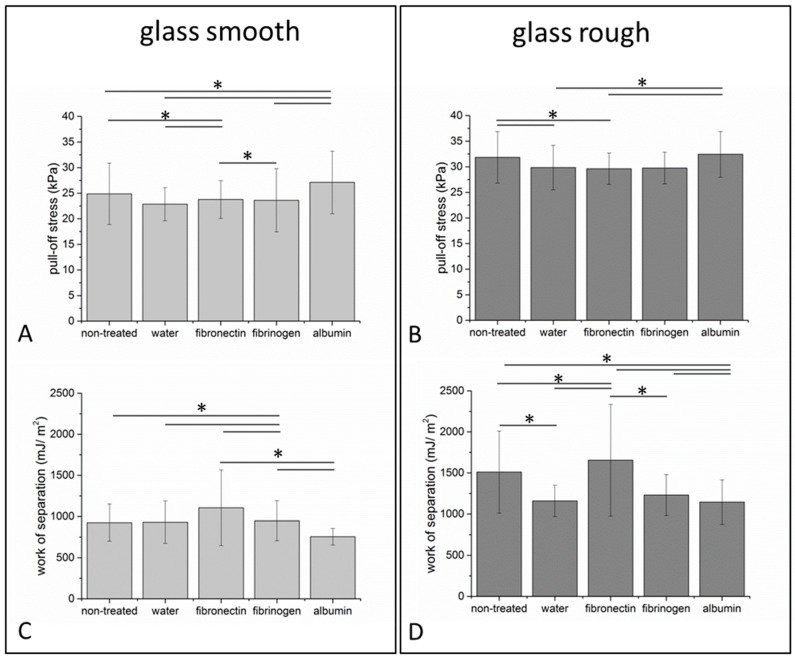
Determination of characteristic adhesion parameters by normal tack analysis. Pull-off stress (**A**,**B**) and work of separation (**C**,**D**) of SSA films were determined with two glass substrates with different surface roughness (glass smooth (**A**,**C**) R_Z_ = 0.12 ± 0.004 µm; glass rough (**B**,**D**) R_Z_ = 2.055 ± 0.017 µm) and constant retraction velocity of 10 µm/s. Films were functionalized with fibronectin, fibrinogen, and albumin and compared to control conditions (films immersed in water or non-treated). All films exhibited a thickness of 170 ± 30 µm. Comparable pull-off forces and work of separation were detectable within each group. Number of independent experiments: n = 3. * indicates *p* ≤ 0.05. Original data is included in Supplementary Table S1.

To answer the question to which extent the functionalization of SSA films influences the macroscopic adhesion process, tack analysis was performed with two substrates possessing different surface roughness, as shown in Figure 4. All three protein coatings were analyzed in relation to pristine polymers and polymers immersed in ddH_2_O for 1 h at 37 °C. In all cases, higher substrate roughness resulted in statistically significant higher pull-off stress and work of separation, as shown in Figure 4. For example, focusing on the non-treated conditions, the pull-off stress increased by about 20% on the rough samples compared to the smooth sample, while the work of separation was approximately 60% higher. This increase might be explained by crack trapping effects due to the micro-roughness [46,47,48]. As described earlier, SSA exhibits cavitation and fibrillation while the materials elongate by up to 200% [22].

Especially for albumin and fibronectin, statistically significant effects on the adhesive performance were observed, as shown in Figure 4. A pull-off stress of 27.11 ± 6.1 kPa was detected on albumin coated surfaces with the smooth substrate, which is different to the values measured on pristine surfaces (24.87 ± 6 kPa) and also statistically different from films immersed solely in ddH_2_O (22.9 ± 3.2 kPa), as shown in Figure 4. A work of separation of 755.5 ± 100 mJ/m^2^ was measured with the smooth glass substrate on albumin functionalized films, demonstrating no significant difference to the control conditions (926 ± 225.9 mJ/m^2^ for pristine and 931.9 ± 257.6 mJ/m^2^ for H_2_O), as shown in Figure 4. Statistically significant differences were also observed for fibronectin treated surfaces and both control conditions, as shown in Figure 4. In contrast to albumin and fibronectin, we could not detect any significant influence of fibrinogen on the pull-off stress, as shown in Figure 4.

It is commonly assumed that van der Waals forces play an essential role in adhesion [49]. With an attractive potential proportional to 1/*r*^6^, where *r* is the distance between the interacting atoms, an intimate contact in the micro to nano range is necessary to achieve a notable attraction [50,51]. Therefore, interaction forces between two contacting materials can be positively or negatively affected by deposited particles or molecules between them [52,53,54]. Roughness and film thickness are critical mediators of the adhesive performance of soft elastomers [9]. We observed before, that for any application related to skin and tissue adhesion, the thickness of the adhesive layer should be taken carefully into account in relation to the surface roughness. Therefore, we selected a film thickness of 170 ± 30 µm, showing optimal adhesive performance on surface with a roughness up to Rz = 50 µm, being close to the roughness of human skin as well [9]. Here, a rough glass substrate with an Rz of 2.055 ± 0.017 µm has been selected, because we expected the roughness of the tympanic membrane to be smaller than values obtained for skin roughness and higher than the Rz = 0.12 ± 0.004 µm of the smooth glass.

Our results indicate that the adhesive performance was strongly affected by surface roughness. To the contrary, protein treatment induced only a slight influence on the adhesive properties of the films on both substrates. Focusing on the rough substrate and only comparing the water condition and the protein treated surfaces, we observed a significantly higher pull-off stress for albumin, while no influence was detected for fibrinogen and fibronectin. We conclude that the adhesion, determined by tack analysis, was not strongly influenced by protein treatment.

### 3.4. Tack and Peel Analysis of Protein Functionalized Composite Films

**Figure 5:**
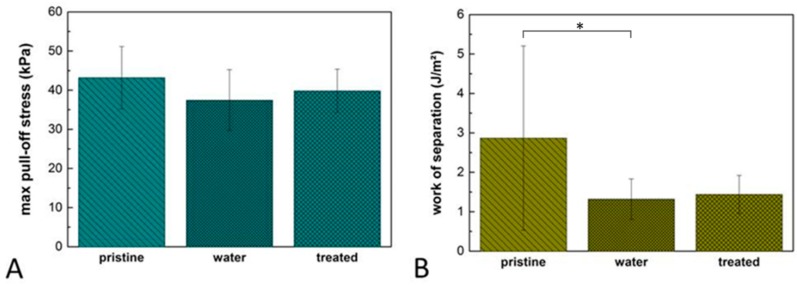
Normal tack analysis of composite films. The films consist of a Sylgard 184 backing layer with a thickness of 43 ± 11 µm and SSA layer exhibiting a thickness of 157 ± 22 µm. Max pull-off stress (**A**) and work of separation (**B**) were determined using a rough glass substrate. Number of independent experiments n = 3. Original data is included in Supplementary Table S2. * indicates *p* ≤ 0.05.

Due to the outstanding ability of fibronectin to promote cellular adhesion and spreading, as shown in Section 3.2, the tack and peel testing experiments with a rough substrate were restricted to fibronectin treated composite films and control conditions. A maximum pull-off stress of 39 ± 6 kPa was detected on fibronectin treated films, as shown in Figure 5. Comparable values were observed on non-treated films (43 ± 8 kPa) or films incubated in water (37 ± 8 kPa), as shown in Figure 5A. Statistical analysis revealed no significant difference while comparing all three conditions to each other, as shown in Figure 5A. The work of separation of the water immersed samples (1.3 ± 0.5 J/m^2^) and the fibronectin treated films (1.4 ± 0.5 J/m^2^) was significantly lower compared to 2.9 ± 2.3 J/m^2^ determined on the pristine film, as shown in Figure 5B.

**Figure 6:**
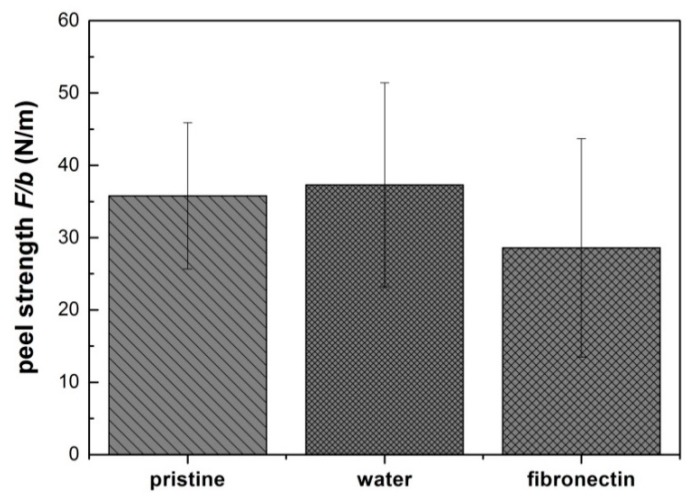
Maximum strength during peel test (F/b) of composite films on a rough glass substrate. (F/b): F indicates the peel force; b indicates the width of the film (0.5 cm). Three conditions were investigated: pristine, stored in ddH_2_O, and coated with fibronectin. The polymeric films were cut into small strips with the dimensions 2 cm × 0.5 cm. During the measurements, the peeling angle decreased from a starting angle of *Ѳ* ≈ 90° to *Ѳ* ≈ 70°–80°. Number of independent experiments n = 3. Original data is included in Supplementary Table S3. Statistical analysis revealed no difference between the three conditions.

While the tack analysis performed with a cylindrical flat-ended probe imposes a well-defined loading on the adhesive film, it does not provide detailed information on the steady state crack propagation during detachment [55]. Therefore, peel tests were performed with the composite films on a glass substrate exhibiting similar roughness as the substrate used for the tack analysis, as shown in Figure 6.

A maximum peel strength of 36 ± 10 N/m was detected on the pristine films, which was comparable to the water treated films (37 ± 14 N/m). A decrease of the peel strength to 29 ± 15 N/m was observed on the fibronectin treated films, as shown in Figure 6. No statistically significant difference was observed while comparing the three conditions.

The film de-bonding process from the substrate can occur by different mechanisms and can involve an extensive deformation in the bulk region of the film, significantly affecting the energy dissipation [55]. Peel tests are usually performed at angles *Ѳ* = 90° or *Ѳ* = 180°, where the force and work of detachment usually increases with increasing *Ѳ* [56]. In our experiments, films were peeled at a constant velocity of 50 µm/s and at a starting angle of *Ѳ* = 90°. Towards termination of the peel off process, *Ѳ* decreased typically to approximately 80° and 70°. We concluded that the adhesive performance of the composite films analyzed with tack tests were comparable to the single layer films investigated in Section 3.3 and a slight, but statistical non-significant decrease in peel strength was detectable after fibronectin coating.

### 3.5. Peel Analysis of Protein Functionalized Composite Films on Explanted Tympanic Membranes

**Figure 7:**
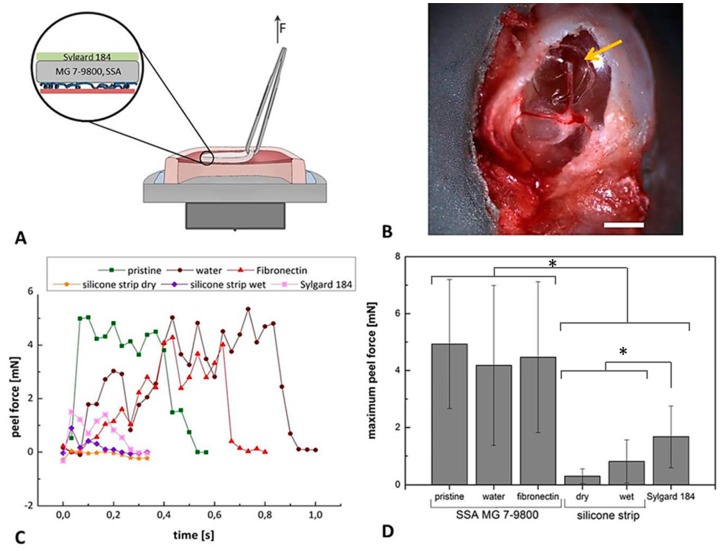
Peel analysis of composite films attached to isolated murine tympanic membranes. For the ex vivo peel tests, tympanic membranes with surrounding bone structures were affixed on a load cell as schematically shown (**A**). The circular films with a diameter of 1.2 mm were manually attached to the tissue (arrow in (**B)**). Peel adhesion of the fibronectin functionalized SSA films were compared to pristine and ddH_2_O treated films. Wet and dry silicone strips and a Sylgard 184 thin film were included as further references. Exemplary peel force analysis for each condition is displayed in (**C)**. The maximum peel forces of all analyzed conditions are presented in (**D**). Number of independently performed analyses n = 3. Scale bar in B represents 1 mm. Original data is included in Supplementary Table S4. * indicates *p* ≤ 0.05.

To test the adhesive properties of composite films on tissue, isolated murine tympanic membranes were chosen as model system, as shown in Figure 7A,B. Peeling of the composite films with a nearly constant velocity indicated a strong attachment of the SSA samples to tissue in contrast to commercially available silicone strips, as shown in Figure 7C. We also used Sylgard 184 films, exhibiting comparable conformation abilities as the SSA-based patches but lacking the adhesive properties of the SSA as a further control. Quantitative analysis indicates significantly improved adhesion of the SSA-based patches compared to conventional silicone strips, as shown in Figure 7D. A maximum peel force of 1.7 ± 1.0 mN was observed for Sylgard 184, significantly higher than the values measured for silicone strips (0.3 ± 0.2 mN for the dry state and 0.8 ± 0.7 mN for the wet state), as shown in Figure 7D. All three SSA-based patches (pristine: 4.9 ± 2.2 mN; water: 4.2 ± 2.8 mN; fibronectin: 4.5 ± 2.6 mN) displayed significantly higher maximum peel forces compared to the control conditions without damaging the tympanic membrane during detachment, as shown in Figure 7D.

Here, we investigated the properties of a dry adhesive possessing a sensitive bonding and de-bonding mechanism, allowing reliable long-term attachment and re-positioning during a medical treatment, which will be the subject of further studies.

## 4. Conclusions

We investigated the adhesive performance of self-adhesive elastomeric films for sensitive adhesion to tissue. The results show that the SSA-based films can be functionalized by physical protein adsorption with only a slight impact on the adhesive performance analyzed by tack tests and peel tests using two different substrates. In particular, a fibronectin coating of the SSA surface significantly improves the cellular interaction of L929 murine fibroblasts with the polymeric surface and has been used for further experiments. The composite materials designed in the current study have been successfully attached to murine explanted ear drums and the adhesive performance has been demonstrated with a peel test. The composite films might represent a promising therapeutic option for the treatment of tympanic membrane perforations.

## Supplementary Materials

The following are available online at https://www.mdpi.com/2073-4360/11/6/942/s1, Figure S1: Schematic representation of the tack and peel measurement setup, Figure S2: Exemplary peel measurements performed with SSA patches attached to excised murine tympanic membranes, Figure S3: Cellular response to the protein coating of the elastomer films after seven days of storage in ddH_2_O before cellular seeding, Table S1: Corresponding table to Figure 4, Table S2: Corresponding table to Figure 5, Table S3: Corresponding table to Figure 6, Table S4: Corresponding table to Figure 7

## References

[1] Chen J., Zheng J., Gao Q., Zhang J., Zhang J., Omisore O., Wang L., Li H. (2018). Polydimethylsiloxane (PDMS)-Based Flexible Resistive Strain Sensors for Wearable Applications. Appl. Sci..

[2] Kenry, Yeo J.C., Lim C.T. (2016). Emerging flexible and wearable physical sensing platforms for healthcare and biomedical applications. Microsyst. Nanoeng..

[3] Thap T., Yoon K., Lee J. (2016). Graphite Based Electrode for ECG Monitoring: Evaluation under Freshwater and Saltwater Conditions. Sensors.

[4] Thanawala S.K., Chaudhury M.K. (2000). Surface Modification of Silicone Elastomer Using Perfluorinated Ether. Langmuir.

[5] Kwak M.K., Jeong H.-E., Suh K.Y. (2011). Rational Design and Enhanced Biocompatibility of a Dry Adhesive Medical Skin Patch. Adv. Mater..

[6] Gun Park D., Chul Shin S., Won Kang S., Tae Kim Y. (2005). Development of flexible self adhesive patch for professional heat stress monitoring service. Conf. Proc. IEEE Eng. Med. Biol. Soc..

[7] Jeong S.H., Zhang S., Hjort K., Hilborn J., Wu Z. (2016). PDMS-Based Elastomer Tuned Soft, Stretchable, and Sticky for Epidermal Electronics. Adv. Mater..

[8] Baik S., Lee H.J., Kim D.W., Kim J.W., Lee Y., Pang C. (2019). Bioinspired Adhesive Architectures: From Skin Patch to Integrated Bioelectronics. Adv. Mater..

[9] Fischer S.C.L., Boyadzhieva S., Hensel R., Kruttwig K., Arzt E. (2018). Adhesion and relaxation of a soft elastomer on surfaces with skin like roughness. J. Mech. Behav. Biomed. Mater..

[10] Thomas X. (2003). Silicone Adhesives in Healthcare Applications. Dow Corning Corp..

[11] Yoshida S., Hagiwara K., Hasebe T., Hotta A. (2013). Surface modification of polymers by plasma treatments for the enhancement of biocompatibility and controlled drug release. Surf. Coat. Technol..

[12] Ai H., Lvov Y.M., Mills D.K., Jennings M., Alexander J.S., Jones S.A. (2003). Coating and Selective Deposition of Nanofilm on Silicone Rubber for Cell Adhesion and Growth. Cell Biochem. Biophys..

[13] Bhati R.S., Mukherjee D.P., McCarthy K.J., Rogers S.H., Smith D.F., Shalaby S.W. (2001). The growth of chondrocytes into a fibronectin-coated biodegradable scaffold. J. Biomed. Mater. Res..

[14] Elbert D.L., Hubbell J.A. (1998). Self-assembly and steric stabilization at heterogeneous, biological surfaces using adsorbing block copolymers. Chem. Biol..

[15] Dee C.K., Puleo D.A., Bizios R. (2003). An Introduction to Tissue-Biomaterial Interactions.

[16] Kim D., Herr A.E. (2013). Protein immobilization techniques for microfluidic assays. Biomicrofluidics.

[17] Cunningham J.J., Nikolovski J., Lin- J.J., Mooney D.J. (2002). Quantification of Fibronectin Adsorption to Research Report. Biotechniques.

[18] Kuddannaya S., Chuah Y.J., Lee M.H.A., Menon N.V., Kang Y., Zhang Y. (2013). Surface Chemical Modification of Poly(dimethylsiloxane) for the Enhanced Adhesion and Proliferation of Mesenchymal Stem Cells. ACS Appl. Mater. Interfaces.

[19] Tan S.H., Nguyen N.-T., Chua Y.C., Kang T.G. (2010). Oxygen plasma treatment for reducing hydrophobicity of a sealed polydimethylsiloxane microchannel. Biomicrofluidics.

[20] Jönsson C., Aronsson M., Rundström G., Pettersson C., Mendel-Hartvig I., Bakker J., Martinsson E., Liedberg B., MacCraith B., Öhman O. (2008). Silane–dextran chemistry on lateral flow polymer chips for immunoassays. Lab Chip.

[21] Beal J.H.L., Bubendorfer A., Kemmitt T., Hoek I., Mike Arnold W. (2012). A rapid, inexpensive surface treatment for enhanced functionality of polydimethylsiloxane microfluidic channels. Biomicrofluidics.

[22] Fischer S.C.L., Kruttwig K., Bandmann V., Hensel R., Arzt E. (2017). Adhesion and Cellular Compatibility of Silicone-Based Skin Adhesives. Macromol. Mater. Eng..

[23] Toworfe G.K., Composto R.J., Adams C.S., Shapiro I.M., Ducheyne P. (2004). Fibronectin adsorption on surface-activated poly(dimethylsiloxane) and its effect on cellular function. J. Biomed. Mater. Res..

[24] Donaldson D.J., Mahan J.T. (1983). Fibrinogen and Fibronectin as Substrates for epidermal cell migration during wound closure. J. Cell Sci.

[25] Tersteeg C., Roest M., Mak-Nienhuis E.M., Ligtenberg E., Hoefer I.E., Groot P.G., Pasterkamp G. (2012). A fibronectin-fibrinogen-tropoelastin coating reduces smooth muscle cell growth but improves endothelial cell function. J. Cell. Mol. Med..

[26] Krajewski S., Neumann B., Kurz J., Perle N., Avci-Adali M., Cattaneo G., Wendel H.P. (2015). Preclinical Evaluation of the Thrombogenicity and Endothelialization of Bare Metal and Surface-Coated Neurovascular Stents. Am. J. Neuroradiol..

[27] An Y.H., Stuart G.W., McDowell S.J., McDaniel S.E., Kang Q., Friedman R.J. (1996). Prevention of bacterial adherence to implant surfaces with a crosslinked albumin coatingin vitro. J. Orthop. Res..

[28] Villar-Fernandez M.A., Lopez-Escamez J.A. (2015). Outlook for tissue engineering of the tympanic membrane. Audiol. Res..

[29] Hong P., Bance M., Gratzer P.F. (2013). Repair of tympanic membrane perforation using novel adjuvant therapies: A contemporary review of experimental and tissue engineering studies. Int. J. Pediatr. Otorhinolaryngol..

[30] Kozin E.D., Black N.L., Cheng J.T., Cotler M.J., McKenna M.J., Lee D.J., Lewis J.A., Rosowski J.J., Remenschneider A.K. (2016). Design, fabrication, and in vitro testing of novel three-dimensionally printed tympanic membrane grafts. Hear. Res..

[31] Boyadzhieva S., Fischer S.C.L., Lösch S., Rutz A., Arzt E., Kruttwig K. (2018). Thin Film Composite Silicon Elastomers for Cell Culture and Skin Applications: Manufacturing and Characterization. J. Vis. Exp..

[32] Kroner E., Blau J., Arzt E. (2012). Note: An adhesion measurement setup for bioinspired fibrillar surfaces using flat probes. Rev. Sci. Instrum..

[33] Bundy K., Schlegel U., Rahn B., Geret V., Perren S. (2000). An improved peel test method for measurement of adhesion to biomaterials. J. Mater. Sci. Mater. Med..

[34] Comelles J., Estévez M., Martínez E., Samitier J. (2010). The role of surface energy of technical polymers in serum protein adsorption and MG-63 cells adhesion. Nanomed. Nanotechnol. Biol. Med..

[35] Toworfe G., Composto R., Adams C., Shapiro I., Ducheyne P. (2003). Effect of surface activated poly(dimethylsiloxane) on fibronectin adsorption and cell function. Mat. Res. Soc. Symp. Proc..

[36] Kottke-Marchant K., Anderson J.M., Umemura Y., Marchant R.E. (1989). Effect of albumin coating on the in vitro blood compatibility of Dacron^®^ arterial prostheses. Biomaterials.

[37] Horváthy D.B., Simon M., Schwarz C.M., Masteling M., Vácz G., Hornyák I., Lacza Z. (2017). Serum albumin as a local therapeutic agent in cell therapy and tissue engineering. BioFactors.

[38] Horváthy D.B., Vácz G., Cselenyák A., Weszl M., Kiss L., Lacza Z. (2013). Albumin-Coated Bioactive Suture for Cell Transplantation. Surg. Innov..

[39] Bernards M.T., Qin C., Jiang S. (2008). MC3T3-E1 cell adhesion to hydroxyapatite with adsorbed bone sialoprotein, bone osteopontin, and bovine serum albumin. Colloids Surf. B Biointerfaces.

[40] Yamazoe H., Tanabe T. (2008). Preparation of water-insoluble albumin film possessing nonadherent surface for cells and ligand binding ability. J. Biomed. Mater. Res. Part A.

[41] Kalaskar D.M., Downes J.E., Murray P., Edgar D.H., Williams R.L. (2013). Characterization of the interface between adsorbed fibronectin and human embryonic stem cells. J. R. Soc. Interface.

[42] Peterson S.L., McDonald A., Gourley P.L., Sasaki D.Y. (2005). Poly(dimethylsiloxane) thin films as biocompatible coatings for microfluidic devices: Cell culture and flow studies with glial cells. J. Biomed. Mater. Res..

[43] Katsen-Globa A., Peter L., Zöllner S., Dörge T., Daffertshofer M., Preckel H., Schmitt D., Zimmermann H. (2009). A novel approach for automated analysis of cell attachment and spreading based on backscattered electron imaging by scanning electron microscopy. Materials.

[44] Novotna Z., Reznickova A., Rimpelova S., Vesely M., Kolska Z., Svorcik V. (2015). Tailoring of PEEK bioactivity for improved cell interaction: Plasma treatment in action. RSC Adv..

[45] Zelzer M., Albutt D., Alexander M.R., Russell N.A. (2012). The Role of Albumin and Fibronectin in the Adhesion of Fibroblasts to Plasma Polymer Surfaces. Plasma Process. Polym..

[46] Purtov J., Gorb E.V., Steinhart M., Gorb S.N. (2013). Measuring of the hardly measurable: Adhesion properties of anti-adhesive surfaces. Appl. Phys. A Mater. Sci. Process..

[47] Davis C.S., Martina D., Creton C., Lindner A., Crosby A.J. (2012). Enhanced adhesion of elastic materials to small-scale wrinkles. Langmuir.

[48] Guduru P.R., Bull C. (2007). Detachment of a rigid solid from an elastic wavy surface: Experiments. J. Mech. Phys. Solids.

[49] Owens D.K., Wendt R.C. (1969). Estimation of the surface free energy of polymers. J. Appl. Polym. Sci..

[50] Klatt J., Barcellona P., Bennett R., Bokareva O.S., Feth H., Rasch A., Reith P., Buhmann S.Y. (2017). Strong van der Waals Adhesion of a Polymer Film on Rough Substrates. Langmuir.

[51] Israelachvili J.N., Tabor D. (1972). The measurement of van der Waals dispersion forces in the range 1.5 to 130 nm. Proc. R. Soc. London. A. Math. Phys. Sci..

[52] Persson B.N.J., Tosatti E. (2001). The effect of surface roughness on the adhesion of elastic solids. J. Chem. Phys..

[53] Kendall K., Roberts A.D. (2014). van der Waals forces influencing adhesion of cells. Philos. Trans. R. Soc. B Biol. Sci..

[54] Valle-Delgado J.J., Molina-Bolívar J.A., Galisteo-González F., Gálvez-Ruiz M.J., Feiler A., Rutland M.W. (2004). Interaction Forces between BSA Layers Adsorbed on Silica Surfaces Measured with an Atomic Force Microscope. J. Phys. Chem. B.

[55] Creton C., Ciccotti M. (2016). Fracture and adhesion of soft materials: A review. Reports Prog. Phys..

[56] Gent A.N., Kaang S.Y. (1987). Effect of peel angle upon peel force. J. Adhes..

