# Supplementary material for: A Self-Adhesive Elastomeric Wound Scaffold for Sensitive Adhesion to Tissue
Source: Polymers (Basel). 2019 May 31;11(6):942. doi: 10.3390/polym11060942 (PMC6630294; doi:10.3390/polym11060942)
Supplement: Supplementary file 1 [file polymers-11-00942-s001.pdf]

polymers

Supplementary Materials

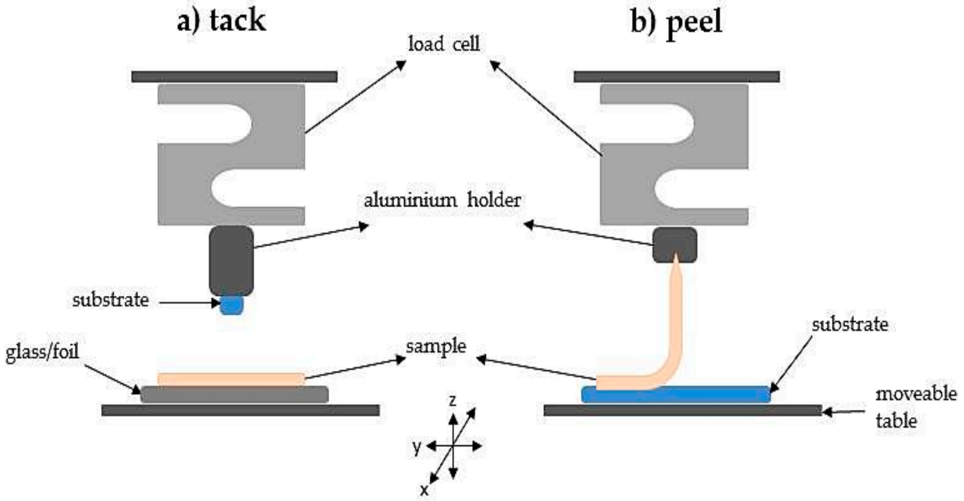

Figure 1. Schematic representation of the tack and peel measurement setup. This graphic depicts the schematic setup for tack tests (**a**) and peel tests (**b**).

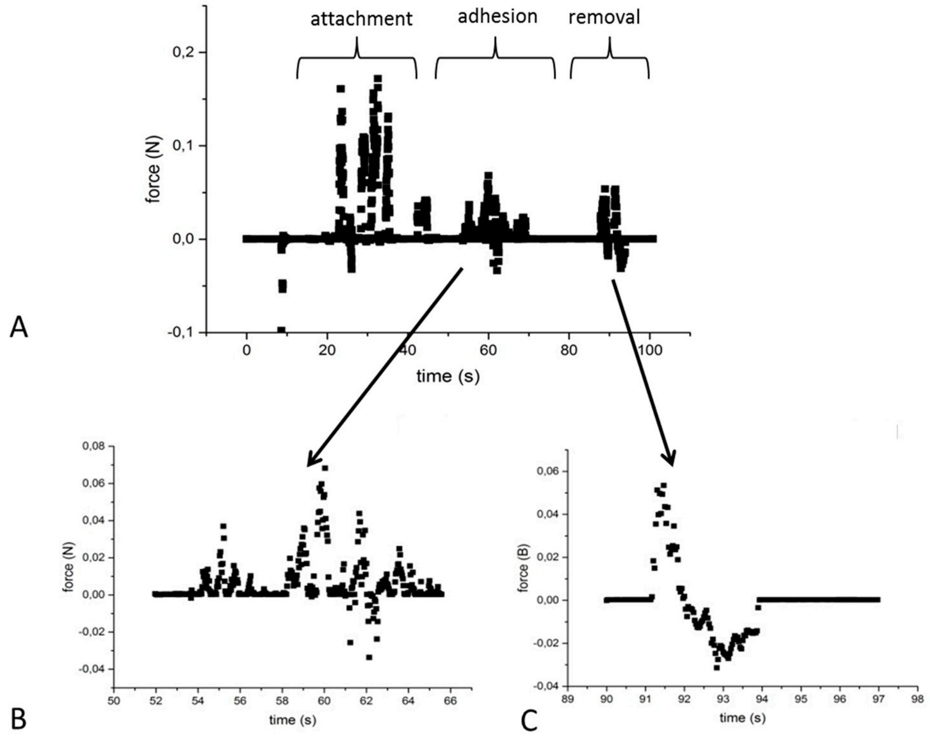

Figure 2. Exemplary peel measurements performed with SSA patches attached to excised murine tympanic membranes. Overview about a typical measurement performed to analyze peel strength of patches on the tympanic membrane (**A**). The measurement consists of three different steps: (1) In the attachment phase, the SSA patch and control materials are applied to the tympanic membrane. In this phase, the contact between patch and tympanic membrane is not uniformly reached. (2) In the adhesion phase, the patches are uniformly adhered to the tympanic membrane by the application of slight pressure all over the entire surface. This phase is separately shown in **B**. (3) In the removal phase, the patch is peeled off the tympanic membrane while trying to maintain a constant speed. This phase is separately shown in **C**. Here, the removal phase is represented by the data points between 92 s and 94 s.

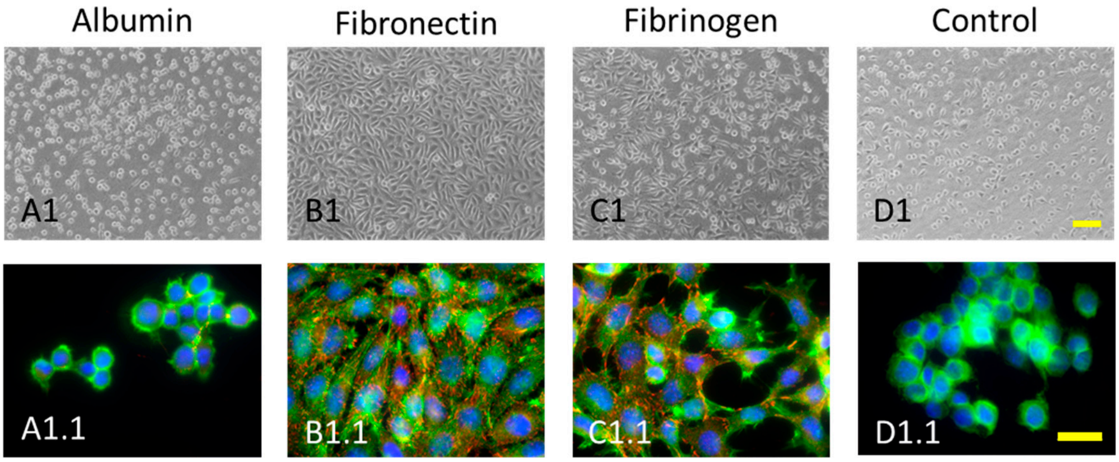

Figure 3. Cellular response to the protein coating of the elastomer films after seven days of storage in ddH_2O before cellular seeding. L929 murine fibroblasts were cultured on SSA films after storage for seven days in water. Before storage, the films were incubated with albumin (**A1**, **A1.1**), fibronectin (**B1**, **B1.1**), and fibrinogen (**C1**, **C1.1**). Controls were non-treated (pristine) films (**D1**, **D1.1**). Phase contrast pictures were taken after a culture period of 48 h (**A1**, **B1**, **C1**, **D1**). Additionally, after fixation, immunocytochemistry was performed with an anti-phospho-FAK^{Tyr397} antibody to visualize focal adhesion contacts (red). Additionally, the actin cytoskeleton (green) and cellular nuclei (blue) were visualized after staining with Alexa 488 conjugated phalloidin and Hoechst Dye 33342 (A1.1, B1.1, C1.1, and D1.1). Number of independent experiments: n = 3 for the fluorescence analysis; n=4 for the determination of the cellular area. Scale bar in A1, B1, C1, D1 = 100 μm; scale bar in A1.1, B1.1, C1.1, D1.1 = 25 μm.

Table S1. Corresponding table to Figure 4.

test parameters: single SSA MG 7-9800 layers on glass

preload stress 13 ± 5 kPa; approach velocity: 30 μm/s; detachment velocity: 10 μm/s; hold time (in contact): 1s

		water	*fibronectin*	*untreated*	*fibrinogen*	*albumin*
pull-off stress [kPa]	**glass smooth**	22.9 ± 3.2	23.8 ± 3.7	24.9 ± 6	23.6 ± 6.2	27.1 ± 6.1
pull-off stress [kPa]	**glass rough**	29.9 ± 4.4	29.6 ± 3.1	31.8 ± 5	29.76 ± 3.1	32.4 ± 4.5
work of separation [mJ/m²]	**glass smooth**	932 ± 256	1106 ± 458	926 ± 226	948 ± 243	756 ± 100
work of separation [mJ/m²]	**glass rough**	1159 ± 191	1655 ± 681	1510 ± 500	1231 ± 250	1145 ± 270

Table S2. Corresponding table to Figure 5.
test parameters: composite films (SSA MG 7-9800 layers and Sylgard 184) on PET foil
preload stress 13 ± 5 kPa; approach velocity: 30 µm/s; detachment velocity: 10 µm/s;
hold time (in contact): 1s

**pull-off stress [kPa]**		**glass rough**		**work of separation [mJ/m²]**		**glass rough**	
	water	*fibronectin*	*untreated*		*water*	*fibronectin*	*untreated*
	37.4 ± 7.8	40 ± 5.5	43 ± 8		1320 ± 512	1438 ± 483	2870 ± 2340

Table S3. Corresponding table to Figure 6.
test parameters: composite films (SSA MG 7-9800 layers and Sylgard 184)
initial peel angle: ≈ 90°; detachment velocity: 10 µm/s

**peel strength [N/m]**		**glass rough**	
	water	*fibronectin*	*untreated*
	373 ± 141	286 ± 151	358 ± 101

Table S4. Corresponding table to Figure 7.
test parameters: composite films (SSA MG 7-9800 layers and Sylgard 184), silicone strip, single layer Sylgard 184. The films were manually applied to the tympanic membrane and peeled off using tweezers.

**maximum peel force [mN]**		**SSA MG 7-9800**		**silicone strip**		**Sylgard 184**
	pristine	*fibronectin*	*water*	*dry*	*wet*	
	4.9 ± 2.3	4.2 ± 2.8	4.5 ± 2.7	0.3 ± 0.25	0.8 ± 0.75	1.7 ± 1.1
